# Preoperative contrast-enhanced CT imaging and clinicopathological characteristics analysis of mismatch repair-deficient colorectal cancer

**DOI:** 10.1186/s40644-023-00591-6

**Published:** 2023-10-12

**Authors:** Shuai Chen, Wenzhe Du, Yuhai Cao, Jixia Kong, Xin Wang, Yisen Wang, Yang Lu, Xiang Li

**Affiliations:** 1 Department of Radiology, The Second Hospital of Dalian Medical University, Zhongshan Road No.467, Shahekou District, Dalian, Liaoning 116023 China; 2 Department of Pathology, The Second Hospital of Dalian Medical University, Zhongshan Road No.467, Shahekou District, Dalian, Liaoning 116023 China

**Keywords:** Microsatellite instability, DNA mismatch repair, Colorectal cancer, Computed tomography, Clinicopathology

## Abstract

**Background:**

Colorectal cancer (CRC) can develop through various pathogenetic pathways, and one of the primary pathways is high microsatellite instability (MSI-H)/deficient mismatch repair (dMMR). This study investigated the correlation between preoperative contrast-enhanced CT (CECT) and clinicopathologic characteristics of colorectal cancer (CRC) according to different mismatch repair (MMR) statuses.

**Methods:**

From April 2021 to July 2022, a total of 281 CRC patients with preoperative CECT and available MMR status were enrolled from a single centre for this retrospective study. Preoperative CECT features and clinicopathologic characteristics were analysed. Univariate and multivariate logistic regression analyses were used for statistical analysis. A nomogram was established based on the multivariate logistic regression results. Preoperative and postoperative dynamic nomogram prediction models were established. The C-index, a calibration plot, and clinical applicability of the two models were evaluated, and internal validation was performed using three methods.

**Results:**

In total, 249 patients were enrolled in the proficient mismatch repair (pMMR) group and 32 patients in the deficient mismatch repair (dMMR) group. In multivariate analysis, tumour location (right-hemi colon vs. left-hemi colon, odds ratio (OR) = 2.90, *p* = .036), the hypoattenuation-within-tumour ratio (HR) (HR > 2/3 vs. HR < 1/3, OR = 36.7, *p* < .001; HR 1/3–2/3 vs. HR < 1/3, OR = 6.05, *p* = .031), the number of lymph nodes with long diameter ≥ 8 mm on CECT (OR = 1.32, *p* = .01), CEA status (CEA positive vs. CEA negative, OR = 0.07, *p* = .002) and lymph node metastasis (OR = 0.45, *p* = .008) were independent risk factors for dMMR. Pre- and postoperative C-statistic were 0.861 and 0.908, respectively.

**Conclusion:**

The combination of pre-operative CECT and clinicopathological characteristics of CRC correlates with MMR status, providing possible non-invasive MMR prediction. Particularly for dMMR CRC, tumour-draining lymph node status should be prudently evaluated by CECT.

## Introduction

Colorectal cancer (CRC) can develop through various pathogenetic pathways, and one of the primary pathways is high microsatellite instability (MSI-H)/deficient mismatch repair (dMMR) [1]. This type of CRC is seen in about 12–15% of cases, both hereditary and sporadic [2]. Standard treatment for advanced CRC is 5-fluorouracil-based therapies [3]. However, it has been shown that the mismatch repair (MMR) status can predict response to adjuvant therapy in early-stage CRC. Specifically, MSI-H/dMMR CRC does not benefit from 5-fluorouracil adjuvant therapy [4]. Recently, PD-1 pathway inhibitors have emerged as highly effective treatment for MSI-H/dMMR CRC, with the potential to improve prognosis [5, 6]. In fact, neoadjuvant immunotherapy is recommended in NCCN guidelines for CRC patients with clinical T4b dMMR/MSI-H. Therefore, accurate assessment of MMR status is crucial for treatment planning, improving outcomes and prognosis.

To determine MMR status in patients, immunohistochemical (IHC) and DNA-based tests can be performed on surgically collected specimens or endoscopic biopsy samples. Preoperative MMR status is important for patients who may require neoadjuvant immunotherapy. However, one of the real-world situations that exist is lack of sufficient biopsy samples to complete IHC or DNA-based tests [7]. For some patients receiving neoadjuvant therapy, postoperative IHC results for MMR protein are unreliable and may not be fully consistent with IHC results from endoscopic biopsies performed before treatment [8–10]. Lynch syndrome, the most common inherited colon cancer syndrome, is associated with germline mutations in one of the MMR genes (MLH1, MSH2, MSH6, or PMS2). MMR status testing is recommended for Lynch syndrome diagnosis and assists in identification of families with the syndrome and notification of family members and relatives of their risk for the disease [11]. Hence, non-invasive methods that can predict MSI-H/dMMR status are a critical need.

Contrast-enhanced computed tomography (CECT) is a commonly used imaging technique for preoperative staging and assessing the efficacy of treatment in CRC patients. We conducted a comprehensive search of studies investigating CECT features of dMMR/MSI-H CRC. However, most studies related to MSI-H/dMMR status in CRC have focused on radiomics or clinicopathological information [12–14]. Radiomics has limited clinical interpretability, and the predictive performance of clinicopathological characteristics is suboptimal. To our knowledge, there exists a study that incorporates quantitative features derived from CECT in CRC [15]. The assessment of these features on CT includes tumour maximum size, T stage, lymph node status, and inflammatory response. Although this study combined radiomics and clinical-pathological features to predict MSI-H/dMMR status, our understanding of CECT features in dMMR/MSI-H CRC remains limited. The ability to non-invasively predict MMR status can help dMMR/MSI-H CRC patients to benefit from PD-1 pathway inhibitors and avoid adjuvant 5-fluorouracil therapy.

This study aimed to investigate preoperative CECT and clinicopathologic characteristics of CRC patients based on MSI/MMR status. The goal was to develop a predictive model that utilizes CECT features and clinicopathological information to differentiate between MSI-H/dMMR CRC and proficient mismatch repair (pMMR) CRC.

## Methods

This retrospective single-centre study was approved by the ethics committee of our hospital, which waived the need for written informed consent.

### Study participants

**Fig. 1:**
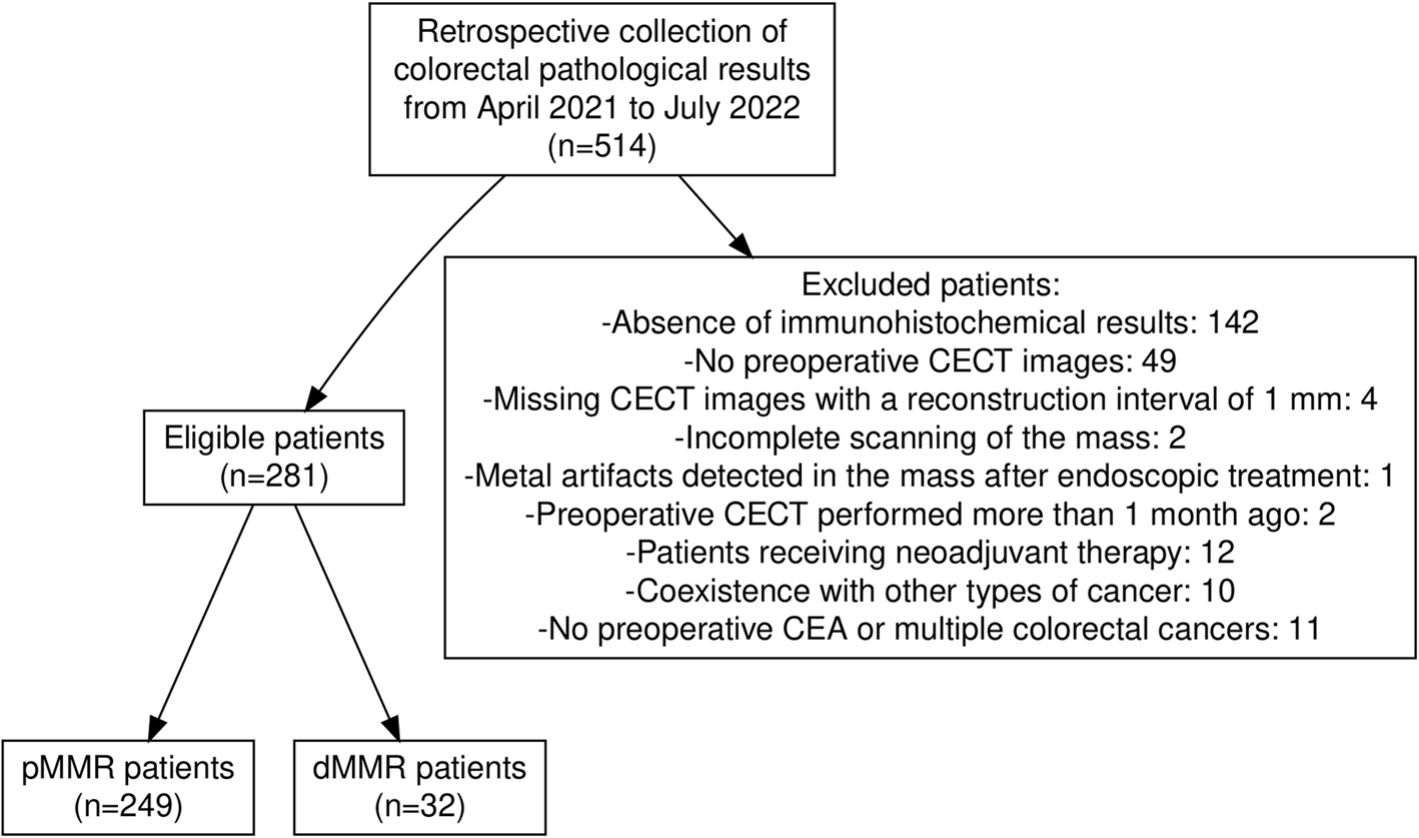
Flow chart of the patient enrolment

From April 2021 to July 2022, we conducted a single-centre retrospective study and collected pathological results for CRC patients at our hospital. The eligibility criteria were as follows: (a) complete MSI/MMR results of the CRC sample; (b) complete preoperative imaging and clinical data. Preoperative imaging examinations included CECT and unenhanced CT. The exclusion criteria were as follows: (a) CECT images could not be obtained, or the quality of CECT images was not satisfactory for diagnosis; (b) combined with other types of cancer or neoadjuvant treatment; (c) missing pre-surgical CEA or multiple CRCs. Figure 1 provides the specific numbers of individuals included and excluded.

### Clinicopathological characteristics of patients

Clinical characteristics including age, sex, carcinoembryonic antigen (CEA), alpha fetoprotein (AFP), and carbohydrate antigen (CA) 125, CA153, CA199, and CA242 were recorded. CEA positivity was defined as greater than or equal to 5 ng/ml; CEA negativity was defined as less than 5 ng/ml. The pathological characteristics recorded included the degree of tumour differentiation, presence of mucinous component (MC), perineural invasion (PI), lympho-vascular invasion (LVI), peritoneal metastasis (PM), tumour deposit (TD), the number of lymph node (LN) metastasis, and the number of LN yields. The LN metastasis ratio was calculated as the number of LNs metastasized over the number of LN yields.

The study also determined the location of CRC based on surgical records, endoscopic findings, and CECT. CRCs were classified as either located in the right hemi-colon or left hemi-colon, with the distal one-third of the transverse colon, descending colon, sigmoid colon, and rectum included in the left-hemi colon and the cecum, ascending colon, and proximal two-thirds of the transverse colon included in the right-hemi colon.

### Imaging protocol

A CT scanner with 128 detector rows was used for this research (Philips Brilliance iCT 256, Royal Dutch Philips Electronics Ltd). CT examinations included both unenhanced and the enhanced images. The acquisition parameters were as follows: tube current of 224 mAs, tube voltage of 120 kVp, collimation 128.0 × 0.625 mm, increment 0.5–3 mm and slice thickness of 1 to 3 mm. The contrast agent used was VISIPAQUE (320 mg/ml, GE Healthcare Ireland Limited), administered at a syringe rate of 3.0 ml/s, with a total dose ranging from 75 to 150 ml. Arterial and venous phases were obtained at 25–35 s and 55–75 s after injection of the contrast agent, respectively. All scans were performed from the top of the diaphragm to the distal end of the rectum. First, a non-enhanced CT scan was performed to obtain baseline images, followed by administration of a contrast agent to assess the drug concentration in the abdominal aorta. Upon reaching the threshold, arterial phase and venous phase scans were initiated. The images acquired were then uploaded to the picture archiving and communication system (PACS) system.

### Imaging analysis

CT images were independently evaluated by two radiologists, one with over ten years of experience in abdominal diagnosis (X.L.) and the other with five years of experience (S.C.). The two radiologists adjusted the window width and level to 150 and 50, respectively, and compared the non-enhanced CT and enhanced CT images of CRC. In case of any disagreement, the two radiologists discussed and reached an agreement. The radiologists were blinded to the clinicopathological features. Multiplanar reformation (MPR) images were used to observe and measure imaging features. Reconstruction was performed using a section thickness of 1 mm and a reconstruction interval of 1 mm. The imaging features assessed included:

Tumour enhancement degree: defined as hyper-/isoenhancement or hypoenhancement compared to the adjacent colon.

**Fig. 2:**
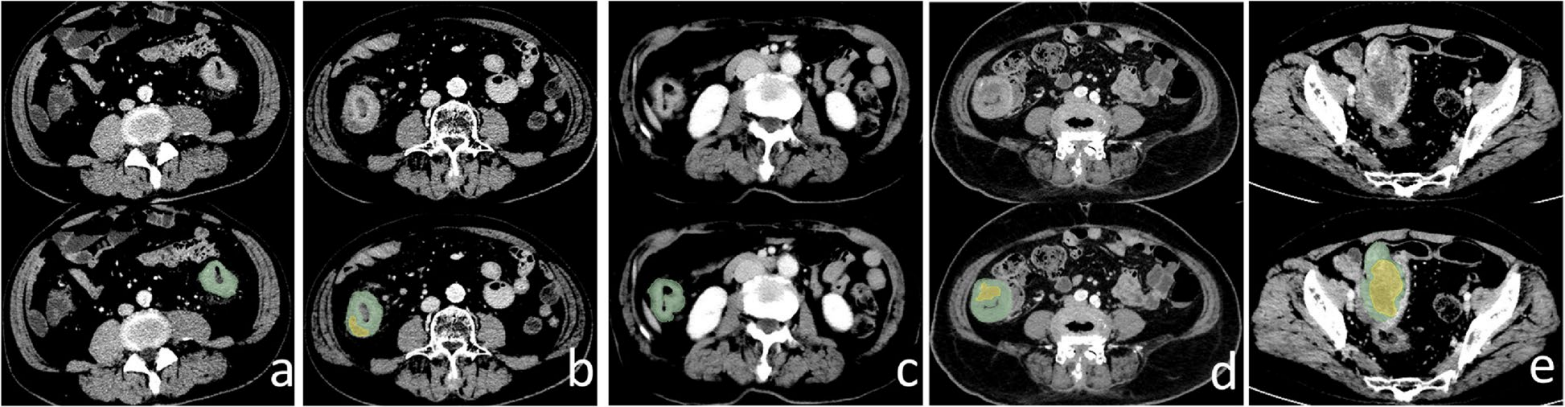
**a, b** The tumour is denoted by green. Green represents the homogeneous portion. The inhomogeneous part of the tumour is denoted by yellow. **a** Homogeneous. **b** Inhomogeneous; **c**, **d**, **e** The tumour is denoted by green. Hypoattenuation-within-tumour is denoted by yellow. **2** Hypoattenuation-within-tumour ratio < 1/3; **d** Hypoattenuation-within-tumour ratio 1/3–2/3; **e** Hypoattenuation-within-tumour ratio > 2/3

Tumour enhancement pattern: defined as homogeneous or inhomogeneous (Fig. 2a, b).

The hypoattenuation-within-tumour ratio (HR): defined as the proportion of poor or no enhancement within the tumour on CECT. HR was categorized as < 1/3, 1/3–2/3, or > 2/3 (Fig. 2c, d, e).

Long and short diameters of the largest LN: measured using MPR images.

The number of LNs: the number of regional LNs with a long diameter (LD) greater than or equal to 5 mm, 8 mm, and 10 mm were counted in venous phase MPR images.

When there was discordance in the “hypoattenuation-within-tumour ratio” feature, we employed the approach depicted in Fig. 2, outlining the tumour and hypoattenuation-within-tumour areas on axial images and comparing the proportion of the hypoattenuation-within-tumour area to the total tumour area. For the “tumour enhancement degree” and “tumour enhancement pattern” features, when there was discordance, we measured the CT values for comparison. When there was a discrepancy in lymph node count or the maximum size of lymph nodes, both radiologists reviewed the lymph node status together and reached a consensus.

### Pathological review and microsatellite instability status analysis

Gross and microscopic examination of resected CRC tissue was conducted in accordance with the 8th edition of AJCC Cancer Staging Manual. The longest and perpendicular lengths of the resected specimen were used to record the long and short tumour sizes, respectively. The tumour size (area) in cm2 was calculated by multiplying the longest length by the perpendicular length.

To evaluate MSI/MMR status, IHC staining of MMR gene proteins (MLH1, MSH2, MSH6, and PMS2) was performed. Two groups were formed based on staining results: a pMMR group, with positive staining of all four MMR proteins, and a dMMR group, lacking any MMR protein staining.

### Statistical analysis

Categorical variables are presented as numbers and percentages. Relationships for categorical data were assessed using Pearson chi-square, continuity corrected chi-square, and Fisher exact tests. Continuous variables are expressed as median (range or interquartile range (IQR)) and compared using the non-parametric test (Mann–Whitney U test). Statistical significance was determined by a two-sided *p* value of < 0.05. Parameters with *p* values < 0.10 were further evaluated using univariate binary logistic regression and backward stepwise multivariate logistic regression (MLR). To ensure model fit and prevent overfitting, the backward stepwise MLR was conducted based on the Akaike information criterion (AIC) by selecting the model with the lowest AIC. Based on the MLR results, pre- and post-operative nomograms were developed to predict risk of dMMR. Five-fold cross-validation, leave-one-out cross-validation, and bootstrapping were used for internal validation of the model. Agreement between the predictions of the model and the actual results was evaluated using the C-statistic. The clinical applicability and net benefit of the model were assessed using decision curve analysis (DCA). Spearman correlation analysis was used to analyse the correlation between clinicopathological and imaging features. R (version 4.2.1), SPSS (version 22.0), and Python (version 3.7.6) were used for statistical analysis and graphing.

## Results

### Study participants

The clinical and pathological characteristics of the CRC patients enrolled are shown in Table 1. We collected pathological results for 514 consecutive patients from April 2021 to July 2022, and finally included 281 patients in our study who met the inclusion and exclusion criteria. Among them, 249 patients were enrolled in the pMMR group and 32 in the MSI-H/dMMR group (Fig. 1).

**Table 1:** Patient demographics and tumour characteristics

Clinicopathological features	dMMR	pMMR	*P*
No. of patients	32(11%)	249(89%)	
Age(IQR, range)	65(19,38–92)	67(12,21–95)	0.116
Sex			0.341
Male	16(50%)	149(60%)	
Female	16(50%)	100(40%)	
Tumour location			**0.001**
Right	22(69%)	90(36%)	
Left	10(31%)	159(64%)	
Differentiation grade			< 0.001***
High	1(3%)	8(3%)	
Moderate	10(35%)	200(80%)	
Low	7(24%)	27(11%)	
Mucinous adenocarcinoma	11(38%)	14(6%)	
Mucinous component, MC			** < 0.001**
Yes	16(50%)	33(13%)	
No	16(50%)	216(87%)	
T stage			0.958***
T1	0	7(3%)	
T2	3(9%)	24(9%)	
T3	25(78%)	179(72%)	
T4	4(13%)	39(16%)	
N stage			0.013***
N0	25(78%)	143(57%)	
N1 + N2	7(22%)	106(43%)	
M stage			1.000**
M0	30(94%)	230(92%)	
M1	2(6%)	19(8%)	
Stage			0.319***
I	3(9%)	28(11%)	
II	20(63%)	112(45%)	
III	7(22%)	90(36%)	
IV	2(6%)	19(8%)	
Perineural invasion, PI			0.216
Positive	6(19%)	77(31%)	
Negative	26(81%)	172(69%)	
Lympho-vascular invasion, LVI			0.341
Positive	10(31%)	102(41%)	
Negative	22(69%)	147(59%)	
Peritoneal metastasis, PM			1.0**
Positive	1(3%)	9(4%)	
Negative	31(97%)	240(96%)	
Tumour deposit, TD			1.0**
Positive	3(9%)	26(10%)	
Negative	29(91%)	223(90%)	
Pre-surgical serum tumour marker(IQR, range)			
CEA(ng/ml)	2.06(2.64,0.35–60)	2.81(8.65,0.2–300.0)	0.110
AFP(ng/ml)	1.76(1.15,1.00–16.00)	1.77(1.83,1.00–19.40)	0.724
CA125, (U/ml)^a^	5.65(7.88,3.00–76.10)	5.01(4.90,2.00–114.90)	0.344
CA153, (U/ml)^b^	3.83(1.07,3.59–17.76)	3.89(1.55,3.50–54.74)	0.328
CA199, (U/ml)^c^	11.14(17.38,3.50–305.47)	11.06(18.71,1.91–467.31)	0.931
CA242, (U/ml)^d^	3.69(8.35,1.00–172.07)	3.57(6.26,1.00–200.00)	0.805
CEA status			**0.014**
Positive	4(12%)	85(34%)	
Negative	28(88%)	164(66%)	
No. of lymph node metastasis (IQR, range)	0(0,0–2)	0(2,0–21)	**0.008**
LN yields (IQR, range)	20(15.75,9–55)	20(14,2–64)	0.071
LN metastasis ratio (IQR, range)	0(0,0–0.1)	0(0.1,0–1)	**0.003**
Tumour long axes in the gross specimen(cm) (IQR, range)	6.75(3.88,2.5–11.5)	4.50(2.50,1.0–12.0)	** < 0.001**
Tumour short axes in the gross specimen(cm) (IQR, range)	5.00(2.38,1.5–9.0)	3.50(2.00,0.9–9.0)	** < 0.001**
Maximum tumour area(cm^2^) (IQR, range)	34.88(32.83,4.5–103.5)	15.20(16.00,1.0–108.0)	** < 0.001**

*P*-values are calculated from Mann–Whitney U test for continuous variables

*LN* Lymph node

^*^P-values are calculated from chi-square test

^**^continuity corrected chi-square test

^***^Fisher exact test for categorical variables

^a^2 cases miss CA125 in pMMR

^b^9 cases miss CA153 in pMMR

^c^4 cases miss CA199 in pMMR

^d^12 cases miss CA242 in pMMR

### Clinical pathological information

Our analysis revealed that dMMR was predominantly found in the right hemi-colon (69%, *p* = 0.001). In terms of differentiation grade, dMMR tumours were more likely to be low grade (24%) and MA (38%) than were pMMR tumours (*p* < 0.001). Moreover, dMMR tumours had a higher incidence of MC than pMMR tumours (*p* < 0.001). The incidence of dMMR was higher in N0 tumours (78%) than in pMMR tumours (*p* < 0.013). Compared to pMMR patients, dMMR patients were mainly CEA negative (88%,* p* < 0.014). The number of LN metastasis in the dMMR group was less than that in the pMMR group (*p* < 0.008). The dMMR group had a lower LN metastasis ratio than the pMMR group (*p* < 0.003). In the dMMR group, the long and short axes of the tumour in the gross specimen were longer than those in the pMMR group (*p* < 0.001). The maximum tumour area in the dMMR group was larger than that in the pMMR group (*p* < 0.001) (Table 1).

We found no differences in other clinicopathological characteristics between the dMMR and pMMR groups (*p* > 0.05).

### CT imaging features

Compared to pMMR tumours, dMMR tumours were more likely to exhibit hypoenhancement (50%, *p* < 0.001) and inhomogeneity (66%, *p* < 0.001) and were more likely to have a high HR (*p* < 0.001). Significant differences were observed between the dMMR and pMMR groups with regard to the largest LN short diameter (*p* < 0.014) and number of LNs with LD ≥ 8 mm (*p* < 0.016) (Table 2).

**Table 2:** Comparison of imaging parameters between dMMR and pMMR colorectal cancer

Imaging features	dMMR	pMMR	*P*
No. of imagings	32	249	
Tumour			
Enhancement degree			** < 0.001**
Hyperenhancement or isoenhancement	16(50%)	220(88%)	
Hypoenhancement	16(50%)	29(12%)	
Enhancement pattern			** < 0.001**
Inhomogenous	21(66%)	65(26%)	
Homogenous	11(34%)	184(74%)	
Hypoattenuation-within-tumour ratio			< 0.001***
< 1/3	16(50%)	229(92%)	
1/3–2/3	5(16%)	8(3%)	
> 2/3	11(34%)	12(5%)	
Lymph node			
Largest LN short diameter (IQR, range)	7.0(2.0,4.00–11.0)	6.0(3.0,3.0–16.0)	**0.014**
Largest LN long diameter (IQR, range)	9.0(5.0,4.00–19.00)	8.0(5.0,3.0–24.0)	0.116
No. of LNs ≥ 5 mm (IQR, range)	6.0(5.0,0–15)	4.0(6.0,0–35)	0.074
No. of LNs ≥ 8 mm (IQR, range)	2.00(4.0,0–7)	1.00(2.0,0–15)	**0.016**
No. of LNs ≥ 10 mm (IQR, range)	0.5(2.0,0–4)	0(1.0,0–7)	0.177

*P*-values are calculated from chi-square test for categorical variables and Mann–Whitney U test for continuous variables

*LN* Lymph node, *No. of LNs* Number of lymph nodes with long diameter

There was no evidence of differences between the dMMR and pMMR groups in the other imaging features (*p* > 0.05).

### Univariate and multivariate logistic regression analyses

Univariate analysis of clinical characteristics and CT features revealed that location, differentiation grade, MC, CEA status, N stage, number of LN metastasis, tumour long axes, tumour short axes, maximum tumour area, enhancement of degree, enhancement of pattern, and HR were significant predictors of dMMR status (*p* < 0.05). The other characteristics did not show a significant association with dMMR status in univariate analysis (Table 3).

**Table 3:** Univariate and multivariate analysis of influencing factors (Logistic regression)

Characteristic	Univariable					Multivariable				
	N	Event N	OR^a^	95% CI^a^	*p*-value	N	Event N	OR^a^	95% CI^a^	*p*-value
Tumour location										
Left-hemi colon	169	10	—	—		169	10	—	—	
Right-hemi colon	112	22	3.89	1.81, 8.92	< 0.001	112	22	2.90	1.10, 8.19	0.036
Differentiation grade										
Moderate	213	13	—	—						
High	9	1	1.92	0.10, 11.7	0.55					
Low	34	7	3.99	1.40, 10.7	0.007					
Mucinous adenocarcinoma	25	11	12.1	4.58, 32.3	< 0.001					
Mucinous component										
No	232	16	—	—						
Yes	49	16	6.55	2.98, 14.5	< 0.001					
N stage										
N0	168	25	—	—						
N1 + N2	113	7	0.38	0.15, 0.86	0.029					
CEA	281	32	0.98	0.94, 1.00	0.16					
CEA status										
Negative	192	28	—	—		192	28	—	—	
Positive	89	4	0.28	0.08, 0.73	0.019	89	4	0.07	0.01, 0.31	0.002
No. of lymph node metastasis	281	32	0.57	0.31, 0.84	0.024	281	32	0.45	0.21, 0.70	0.008
LN positive ratio	281	32	0.00	0.00, 0.03	0.026					
LN yield number	281	32	1.03	1.00, 1.06	0.073					
Tumour long axes in the gross specimen	281	32	1.33	1.14, 1.56	< 0.001					
Tumour short axes in the gross specimen	281	32	1.44	1.19, 1.75	< 0.001					
Maximum tumour area	281	32	1.03	1.01, 1.04	< 0.001					
Enhancement degree										
Hyper-/isoenhancement	236	16	—	—						
Hypoenhancement	45	16	7.59	3.43, 16.9	< 0.001					
Enhancement pattern										
Homogenous	195	11	—	—		195	11	—	—	
Inhomogenous	86	21	5.40	2.52, 12.2	< 0.001	86	21	2.55	0.84, 7.49	0.089
Hypoattenuation-within-tumour ratio										
< 1/3	245	16	—	—		245	16	—	—	
1/3–2/3	13	5	8.95	2.47, 30.2	< 0.001	13	5	6.05	1.20, 33.0	0.031
> 2/3	23	11	13.1	5.00, 34.9	< 0.001	23	11	36.7	8.47, 220	< 0.001
Largest LN short diameter	281	32	1.09	0.95, 1.25	0.19					
CT LN greater or equal to 5 mm	281	32	1.02	0.95, 1.09	0.51					
CT LN greater or equal to 8 mm	281	32	1.10	0.96, 1.25	0.15	281	32	1.32	1.06, 1.64	0.010

*LN* Lymph node, CT LN greater or equal to 8 mm = Number of lymph nodes with long diameter ≥ 8 mm

^a^*OR* Odds Ratio, *CI* Confidence Interval

**Fig. 3:**
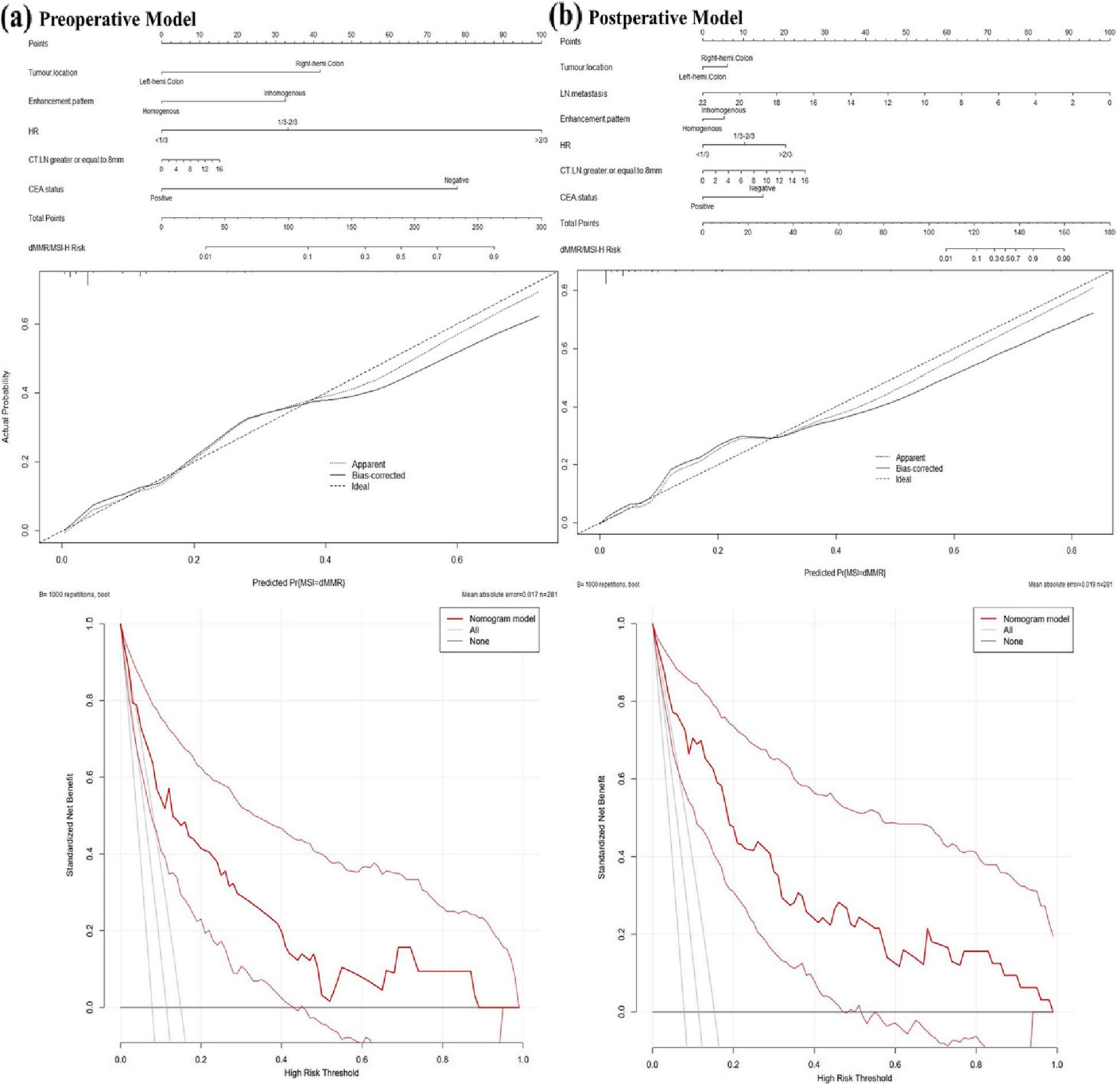
**a** Preoperative model: Nomogram, calibration plot and DCA. **b** Postoperative model: Nomogram, calibration plot and DCA. Note: LN = Lymph node; HR = Hypoattenuation-within-tumour ratio

**Fig. 4:**
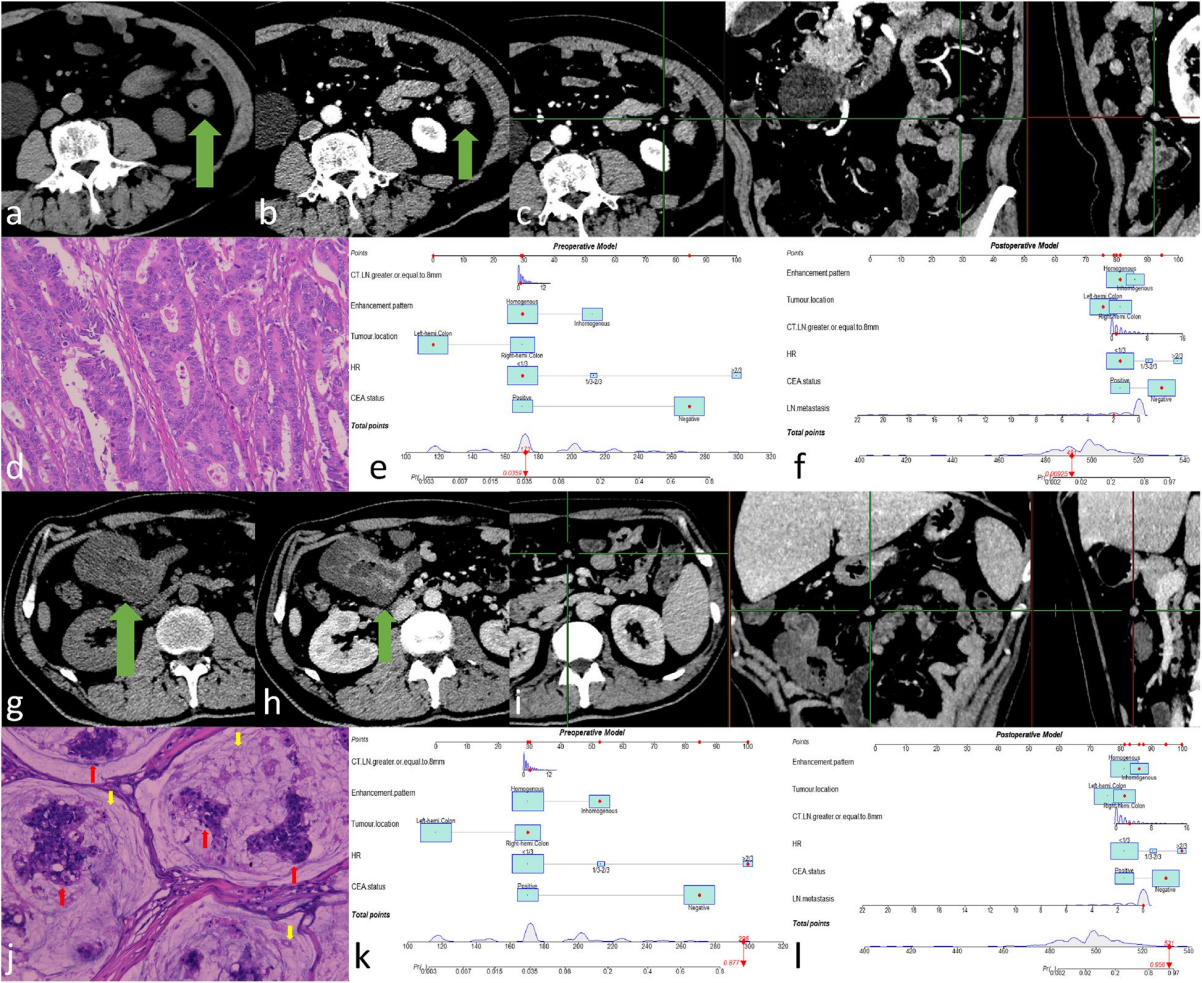
One pMMR CRC case (**a-f**). Male patient, 65 years old. The CEA status was negative. Postoperative pathologic results showed moderately differentiated adenocarcinoma in the left-hemi colon at stage pT4N1M0, with two lymph nodes metastasis. **a** In unenhanced CT images, the tumour appeared as an isoattenuation thickened bowel wall (green arrow). **b** In the venous phase, the tumour as a whole appeared as homogeneous hyperenhancement without hypoattenuation-within-tumour (green arrow). **c** In venous phase MPR images, there was an enhanced lymph node in the local region, with short diameter of 7 mm and long diameter of 8 mm. Only one lymph node had long diameter ≥ 8 mm. **d** After HE staining, the tumour specimen was assessed as moderately differentiated adenocarcinoma. **e**, **f** The pictures show the prediction results of the preoperative model and postoperative model. One dMMR CRC case (**g**-**l**). Male patient, 49 years old. The CEA status was negative. Postoperative pathologic results showed mucinous adenocarcinoma in the right-hemi colon at stage pT4N0M1, with peritoneal metastasis and no lymph node metastasis. **g** In unenhanced CT images, the majority of the tumour appeared as hypoattenuation with 25HU (green arrow). **h** In the venous phase, the tumour as a whole appeared as inhomogeneous enhancement with no enhancement with 29HU in hypoattenuation-within-tumour (green arrow). **i** In venous phase MPR images, there was an enhanced lymph node in the local region, with short diameter of 8 mm and long diameter of 8 mm. Three lymph nodes had long diameter ≥ 8 mm. **j** The tumour specimens were analysed by HE staining. The tumour cell clusters (red arrow) appeared to float in mucinous pools (yellow arrow). **k**, **l** The pictures show the prediction results of the preoperative model and postoperative model

In MLR, MSI-H was significantly associated with location (right hemi-colon versus left hemi-colon, OR:2.90;95% CI:1.10–8.19;*p* = 0.036), CEA status (CEA positive versus CEA negative, OR:0.07;95% CI:0.01–0.31;*p* = 0.002), enhancement of pattern (inhomogeneous versus homogeneous, OR:2.55;95% CI:0.84–7.49;*p* = 0.089), LN metastasis number (OR:1.33;95% CI:1.14–1.56;*p* < 0.001), HR (1/3–2/3 group versus < 1/3 group, OR:6.05;95% CI:1.20–32.99;*p* = 0.031; > 2/3 group versus < 1/3 group, OR:36.73;95% CI:8.47–220.23;*p* < 0.001) and number of LNs with LD ≥ 8 mm (OR:1.32;95% CI:1.06–1.64;*p* = 0.010) (Table 3). Based on the coefficients of MLR, we constructed a postoperative model (Fig. 3) with a C-statistic of 0.908. We further simplified the model by removing number of LN metastases to create a more practical preoperative model (Preoperative-model), which had a C-statistic of 0.861. Calibration of the postoperative model and preoperative model was assessed using calibration plots (Fig. 3). To make use of these nomograms more user friendly, we establish web servers on the Internet (Postoperative-model: https://drcsradiology.shinyapps.io/Postoperative-model-dMMR/; Preoperative-model: https://drcsradiology.shinyapps.io/Preoperative-model-dMMR/) (Fig. 4). The DCA (Fig. 3) and C-statistic indicated that both models were clinically practical, with the postoperative model performing better than the preoperative model.

### Internal validation

The number of validations for leave-one-out cross-validation was equal to the number of all samples. The number of iterations for five-fold cross-validation and bootstrap method was 200. The leave-one-out cross-validation C-statistic results were 0.79 and 0.88 for the pre- and postoperative models, respectively. Using fivefold cross-validation and the bootstrapping method, the preoperative model mean C-statistic result was 0.83 (95% CI: 0.81–0.84) and 0.85 (95% CI: 0.84–0.85), and the postoperative model mean C-statistic results were 0.88 (95% CI: 0.87–0.89) and 0.90 (95% CI: 0.89–0.90) (Table 4).

**Table 4:** Pre- and Post-operative model internal validation results

	Leave-one-out cross-validation	Five-fold cross-validation		Bootstrapping	
	C-statistic	Mean C-statistic	95% CI	Mean C-statistic	95% CI
Preoperative model	0.79	0.83	0.81,0.84	0.85	0.84,0.85
Postoperative model	0.88	0.88	0.87,0.89	0.90	0.89,0.90

### Analysis of correlation

**Fig. 5:**
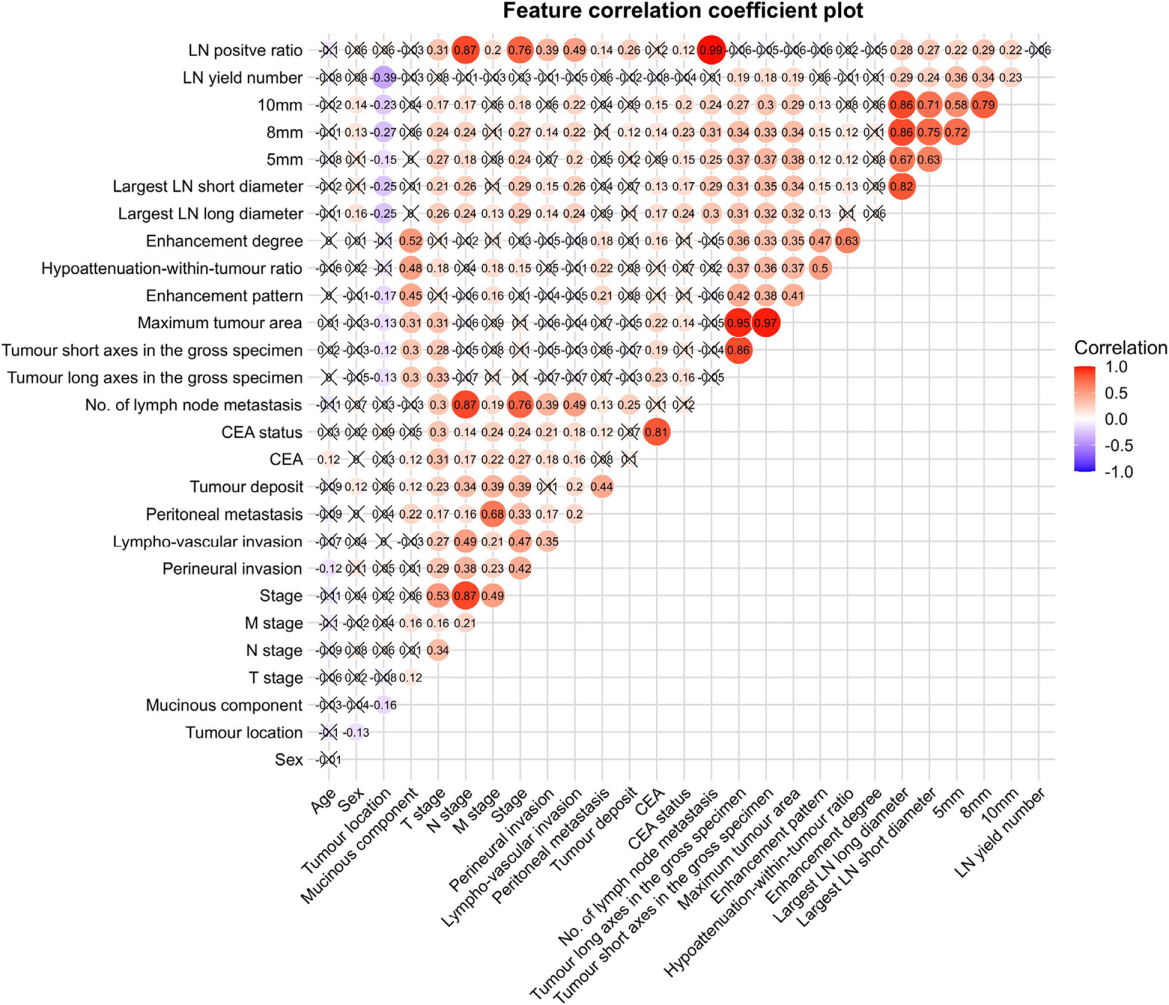
Feature correlation coefficient plot. Note: Categorical variables were treated as dummy variables. Cross marks indicate no statistical significance between the two variables (*p* > .05); LN = Lymph node; 5 mm = Number of lymph nodes with long diameter ≥ 5 mm; 8 mm = Number of lymph nodes with long diameter ≥ 8 mm; 10 mm = Number of lymph nodes with long diameter ≥ 10 mm. Enhancement degree: Hyperenhancement or isoenhancement = 1; Hypoenhancement = 0. Hypoattenuation-within-tumour ratio: < 1/3 = 1; 1/3–2/3 = 2; > 2/3 = 3. Enhancement pattern: Inhomogenous = 1; Homogenous = 0. CEA status: Positive = 1; Negative = 0. Tumour deposit: Positive = 1; Negative = 0. Peritoneal metastasis: Positive = 1; Negative = 0. Lympho-vascular invasion: Positive = 1; Negative = 0. Perineural invasion: Positive = 1; Negative = 0. Mucinous component: Yes = 1; No = 0. Stage: I = 1; II = 2; III = 3; IV = 4. M stage: M1 = 1; M0 = 0. N stage: N1 + N2 = 1; N0 = 0. T stage: T1 = 1; T2 = 2; T3 = 3; T4 = 4. Sex: Male = 1; Female = 2. Tumour location: Right-hemi colon = 1; Left-hemi colon = 2

We conducted Spearman’s correlation coefficient analysis to examine the correlation between all features (Fig. 5). LN yield had a significant positive correlation with the number of LNs on CECT (*p* < 0.05). However, there was no evidence of a correlation between LN yield and the number of LN metastases in our study (*p* > 0.05). We observed a moderate degree of correlation between MC and HR, which was statistically significant (*p* < 0.05).

### Analysis of the hypoattenuation-within-tumour ratio and pathology

We conducted analysis of the proportion of hypoattenuation within tumours and the corresponding pathological results (Table 5). Cross-tabulation of the differentiation degree and mucinous component with HR was assessed using the Fisher exact test at *p*-value < 0.001. In the 1/3–2/3 HR group, the proportion of low differentiation (38.4%) was higher than in other pathological types, and the proportion of pathological samples without mucinous components (53.8%) was higher than that with mucinous components. In the > 2/3 HR group, pathological type mucinous adenocarcinoma accounted for the majority of cases (65.2%), and there were more tumours with MC (73.9%) than without.

**Table 5:** Cross-tabulation of the differentiation degree and mucinous composition with HR

Pathological features	Hypoattenuation-within-tumortumour ratio (HR)			*P*
	HR: < 1/3	HR: 1/3–2/3	HR: > 2/3	
Differentiation grade				< 0.001
Low	27(11.0%)	5(38.4%)	2(8.7%)	
Moderate	204(83.3%)	4(30.8%)	5(21.7%)	
High	8(3.3%)	0(0.0%)	1(4.3%)	
Mucinous adenocarcinoma	6(2.4%)	4(30.8%)	15(65.2%)	
Mucinous component				< 0.001
No	219(89.4%)	7(53.8)	6(26.1%)	
Yes	26(10.6%)	6(46.2%)	17(73.9%)	

## Discussion

In recent years, anti-PD-1 immunotherapy has shown promising results in improving survival for both metastatic and non-metastatic MSI-H/dMMR CRC patients [6]. The potential of MSI/MMR status to guide personalized therapy, predict prognosis, and assess the efficacy of targeted immunotherapy is becoming increasingly recognized.

Our study aimed to analyse differences between dMMR and pMMR CRC in terms of clinicopathological and CT characteristics. Our findings suggest that increased dMMR risk is most highly associated with the right hemi-colon, HR (1/3–2/3 group and > 3/2 group) and the number of LNs with LD ≥ 8 mm. We also found that the dMMR protective factors correlated strongly with CEA positivity and the number of LN metastasis.

Interestingly, the number of LN metastasis served as a protective factor, while the number of LNs with LD ≥ 8 mm on CT played a vital role as a risk factor. For dMMR CRC, we propose that enlarged lymph nodes observed on CT may be attributed to the robust immune response of the primary tumour rather than to lymph node metastasis. This finding is not unprecedented, as previous studies have reported a correlation between lymph nodes and immune response in primary tumours [16–19]. Lal [17] et al. demonstrated that high LN yields in stages II and III colon cancer resection were significantly regulated by broad B- and T-cell adaptive immune responses. Furthermore, MSI-H/dMMR CRC has been shown to have marked “Crohn’s-like” lymphocyte infiltration [2, 20] and tends to have less extensive nodal metastases [21, 22]. These studies suggest that the size and number of LNs may increase in dMMR CRC. However, reactive proliferative LNs and LN metastases are challenging to differentiate on CECT due to similar enhancement patterns and morphological features. Typically, both types exhibit isolated and homogeneous enhancement, often with a round shape. Imaging methods that rely on lymph node size, enhancement pattern and morphological features to estimate the probability of LN metastasis are unreliable and may result in false-positive results, particularly for dMMR CRC. As a result, clinical N stage may be overestimated for dMMR CRC, and lymph nodes should be carefully considered as target lesions to assess the efficacy of chemotherapy or immunotherapy. These findings are crucial for assessing the efficacy of imaging methods for anti-PD-1 therapy and clinical staging.

Although the precise role of PD-1-positive T cells in LNs is still unclear, recent evidence suggests that these cells may play a crucial role in PD-1 blockade-mediated antitumor immunity by enriching tumour-specific progenitor T cells in LNs [23, 24]. In animal models, antitumor immunity is unable to halt tumour progression when lymphocyte migration from LNs is blocked or tumour-draining LNs are dissected [25]. Despite some guidelines recommending dissection of at least 12 lymph nodes, increased LN yield does not increase the number of LN metastases [17]. Our study also suggests that LN yield is not associated with lymph node metastasis (*p* > 0.05). In fact, excessive LN yield might lead to poor prognosis of patients with dMMR CRC [18]. Compared to pMMR CRC, dMMR CRC has a lower tendency for lymph node and distant metastasis [26, 27]. Our MLR results also confirm that LN metastasis is a protective factor for dMMR. Therefore, non-metastatic LN dissection should be carefully considered in dMMR CRC [18, 25]. We believe that combining multi-dimensional information, such as imaging and clinicopathological data, with intraoperative visualization, such as fluorescence molecular imaging [28], will help avoid excessive lymph node dissection in some patients, especially dMMR CRC patients. This approach may be an essential research direction for future studies.

In our research, we found that a tumour in the right hemi-colon was a risk factor in patients with dMMR but that a tumour in the left hemi-colon was a protective factor. The right hemi-colon is more likely to have MCs and shows low differentiation in dMMR CRC. This is consistent with previous studies [2, 29, 30]. It has been proposed that right hemi-colon cancer exhibits higher levels of infiltration of CD4 + T cells and CD8 + T cells than left hemi-colon cancer [31], which is similar to the aforementioned discussion of the relationship between LN and the immune response.

HR was the most significant risk factor for dMMR in our study. Both the mucinous and necrotic components of the tumour show hypoattenuation on CECT. Univariate analysis revealed that MCs and MA were risk factors for dMMR, which was consistent with previous studies [32, 33]. However, MCs and MA were excluded from the MLR analysis, as we believe that HR may be a better predictor for dMMR. Indeed, HR not only reflects the tumour composition but also provides an accurate representation of the degree of mucous or necrosis present.

CRC patients are routinely tested for CEA as a tumour marker for diagnosis and surveillance. In our study, pre-surgical CEA positivity was a protective factor. However, the correlation between CEA and MMR status remains controversial [13, 34, 35]. Based on our study and a review of the existing literature, we found that qualitative analysis of CEA may provide some predictive value for MMR status. However, the reasons why qualitative analysis is superior to quantitative analysis remain unclear. It is possible that quantitative levels of CEA are not associated with the presence of mismatch repair defects but that qualitative judgment of CEA correlates with mismatch repair defects, suggesting that qualitative judgment of CEA has greater value in predicting MMR status. The inconsistencies might be partially attributed to differences in population and sample size. Further research is needed to explore the underlying mechanisms for this observation. Therefore, the association of MMR status with CEA must be interpreted with caution and requires validation using a larger sample size.

Similarly, Zeng [36] et al. conducted a study on preoperative gastric cancer microsatellite instability prediction using imaging and radiomic features, as well as clinical data derived from contrast-enhanced CT. They developed a nomogram based on age, CT-reported N stage, and radiomic score. Although our study also considered clinical indicators, we incorporated additional pathological features such as pathological T stage, N stage, and tumour differentiation, with pathological results as a reference. Furthermore, our study had a larger sample size. Additionally, our research encompassed both qualitative and quantitative investigations. Specifically, we explored the degree of tumour enhancement, the low-density ratio within the tumour, and lymph node involvement. While this study may lack certain quantitative tumour features, we provide detailed analysis of lymph node characteristics. In contrast to Zeng et al., who relied on radiomics to construct a nomogram, our study focused on CT imaging features combined with clinical and pathological data, making it highly applicable and easily replicable for other researchers.

To our knowledge, this is the study to compare clinical, pathological, and CECT features of primary CRC and LNs to predict MSI/MMR status. Our results suggest that in vivo, imaging features of a tumour may be better than clinicopathological features in revealing the characteristics of the tumour itself with regard to some aspects. However, several limitations should be noted. First, this was a retrospective, single-centre study, and the findings need to be confirmed in a large-scale, prospective study. During the study period, we were only able to collect a small number of samples from the dMMR group. Therefore, we aimed to minimize the number of features in our prediction model to ensure reliable results while reducing the risk of overfitting. We are pleased to report that the DCA of our prediction model demonstrated some clinical utility, despite the small sample size of the dMMR group. Additionally, we acknowledge the potential biases that may exist in our study. Patients who received neoadjuvant therapy were excluded from the analysis due to the potential influence of treatment on DNA mismatch repair (MMR) status, as these individuals often present with advanced-stage disease. Furthermore, patients who did not undergo surgery were excluded to avoid inclusion bias resulting from the presence of distant metastasis identified through preoperative imaging or advanced-stage disease that rendered them unsuitable for surgical intervention. Concurrent malignancies were also excluded to minimize potential confounding effects on tumour-related blood markers, such as CEA. Lastly, patients with multiple primary colorectal cancers were excluded to mitigate the impact on N staging caused by the presence of multiple lesions.

## Conclusion

In conclusion, our study suggests that the combination of pre-operative CECT with clinicopathological characteristics of CRC correlates with MMR status, providing a potential possibility for non-invasive MMR prediction. Enlarged LNs on CECT in dMMR CRC may be reactive rather than metastatic. Especially for dMMR CRC, tumour-draining LN status should be prudently evaluated by CECT.

## Data Availability

The datasets during the current study available from the corresponding author on reasonable request.

## Abbreviations

CECT: Contrast-enhanced CT
CRC: Colorectal cancer
MSI-H: High microsatellite instability
MSI: Microsatellite instability
MMR: Mismatch repair
dMMR: Deficient MMR
pMMR: Proficient MMR
IHC: Immunohistochemical
CEA: Carcinoembryonic antigen
AFP: Alpha fetoprotein
CA: Carbohydrate antigen
CA125: Carbohydrate antigen 125
CA153: Carbohydrate antigen 153
CA199: Carbohydrate antigen 199
CA242: Carbohydrate antigen 242
MC: Mucinous component
PI: Perineural invasion
LVI: Lympho-vascular invasion
PM: Peritoneal metastasis
TD: Tumour deposit
LN: Lymph node
PACS: Picture archiving and communication system
LD: Long diameter
MPR: Multiplanar reformation
HR: Hypoattenuation-within-tumour ratio
IQR: Interquartile range
MLR: Multivariate logistic regression
DCA: Decision curve analysis
MA: Mucinous adenocarcinoma
